# Circulating microRNAs as mirrors of acute coronary syndromes: MiRacle or quagMire?

**DOI:** 10.1111/jcmm.12148

**Published:** 2013-11-04

**Authors:** Jin Li, Jiahong Xu, Yan Cheng, Fei Wang, Yang Song, Junjie Xiao

**Affiliations:** 1 Regeneration Lab, School of Life Science, Shanghai UniversityShanghai, China; 2 Experimental Center of Life Sciences, Shanghai UniversityShanghai, China; 3 Department of Cardiology, Tongji Hospital, Tongji University School of MedicineShanghai, China; 4 Department of Psychiatry, Tongji University School of MedicineShanghai, China; 5 Division of Gastroenterology and Hepatology, Digestive Disease Institute, Tongji Hospital, Tongji University School of MedicineShanghai, China

**Keywords:** microRNA, plasma, serum, biomarker, acute coronary syndromes

## Abstract

Acute coronary syndrome (ACS), a leading cause of morbidity and mortality worldwide, is among the most serious cardiovascular diseases. Exploring novel approaches, which can complement and improve current strategies for ACS, is continuous. MicroRNAs (miRNAs) are a novel class of small, short non-coding RNA that post-transcriptionally regulate genes. The tissue- or cell-specific distribution features of miRNAs and its merit of stably existing in serum and plasma make them attractive biomarkers for ACS. An early and accurate diagnosis is the pre-requisite to facilitate rapid decision making and treatment and therefore improve outcome in ACS patients. This review highlights and summarizes recent studies using circulating miRNAs as novel biomarkers for ACS including its role in diagnosis, prediction, prognosis and reaction to therapy. In addition, we also discuss the potential function of miRNAs as extracellular communicators in cell-to-cell communication. Large multicentre studies are highly needed to pave the road for using circulating miRNAs as biomarkers for ACS from the bench to the bedside. Considering the advantageous properties and the continuously increasing number of studies, circulating miRNAs definitely have the potential to be reasonable diagnostic tools once their infancy has passed.

- Introduction
- Diagnostic biomarkers
  – Cardiac and skeletal muscle-specific miRNAs
  – Non-specific cardiac and skeletal muscle miRNAs
- Prediction
- Prognosis
- Reaction to therapy
- Potential biological functions
- Perspective
- Conclusions

## Introduction

Acute coronary syndrome (ACS) is multifactorial, which includes any group of symptoms caused by coronary arteries’ obstruction, ranging from unstable angina (UA), non-ST-segment elevated myocardial infarction (NSTEMI) to ST-segment elevated myocardial infarction (STEMI) 1,2. ACS is among the most serious cardiovascular diseases, making it a leading cause of morbidity and mortality worldwide 3. An accurate and early diagnosis of ACS can definitely help decrease the mortality rate 2. Thus, exploring novel approaches that can complement and improve current strategies for ACS diagnosis and management is important 2.

**Figure 1:**
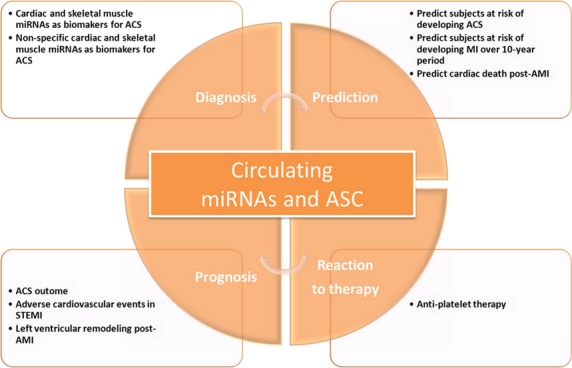
Novel role of circulating microRNAs in acute coronary syndrome. ACS: acute coronary syndrome; AMI: acute myocarial infarction; STEMI: ST-segment elevated myocardial infarction; miR: MicroRNA.

MicroRNAs (miRNAs) are endogenous, 19–25 nucleotide long, short non-coding RNAs that regulate genes post-transcriptionally 4,5. MiRNAs regulate target genes by repressing their translation or inducing their degradation 6. Interestingly, each miRNA can target several mRNAs while each mRNA can be targeted by multiple of miRNAs 5–7. In humans, more than 1000 miRNAs have been identified currently, and the expression of miRNAs appears to exhibit a tissue- or cell-specific distribution 8,9. MiRNAs are crucial for a wide range of essential biological process, including apoptosis, necrosis, autophagy, proliferation, differentiation and development 7–12. Growing evidence has indicated that miRNAs exist in the serum and plasma in a consistent, reproducible and stable manner, opening the possibility of using them as diagnostic surrogate markers for various diseases including cardiovascular disorders 11,13. Moreover, mounting evidence showing that freely circulating miRNAs are informative of human pathology has aroused considerable interest in their diagnostic potential 6–16. However, research on circulating miRNAs is still in its infancy. Accumulating progress has been made in the scenario of using circulating miRNAs as biomarkers for cardiovascular diseases, including ACS, heart failure (HF), diabetes, stroke, essential hypertension and acute pulmonary embolism 17. Among these, ACS is potentially the easiest target to establish a potential role of circulating miRNAs 17. An early and accurate diagnosis is the pre-requisite to facilitate rapid decision making and treatment and therefore improve outcome in ACS patients 1,4. Thus, here we highlight and summarize recent studies using circulating miRNAs as novel biomarkers for ACS, including its role in diagnosis, prediction, prognosis and reaction to therapy (Fig. 1). In addition, we also discuss the potential function of miRNAs as extracellular communicators in cell-to-cell communication.

## Diagnostic biomarkers

### Cardiac and skeletal muscle-specific miRNAs

**Figure 2:**
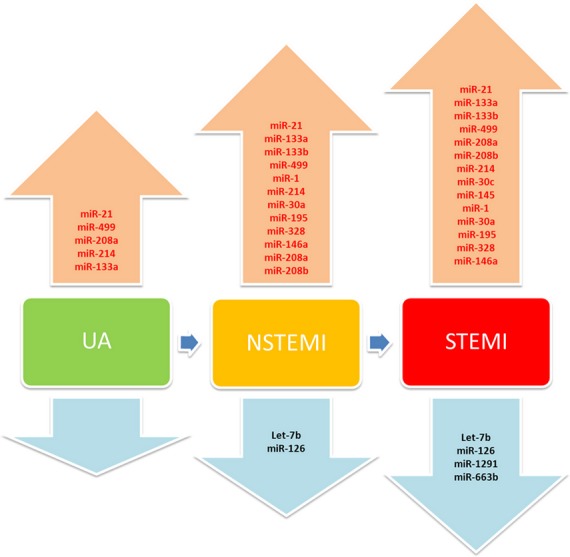
Overview of circulating microRNAs in acute coronary syndrome. UA: unstable angina; NSTEMI: Non-ST- segment elevated myocardial infarction; STEMI: ST-segment elevated myocardial infarction; miR: MicroRNA.

With the initial idea that miRNAs will be released into the circulation from the injured heart, cardiac and skeletal muscle-specific miRNAs including miRNA-1, miRNA-133a, miRNA-133b, miRNA-499, miRNA-208a and miRNA-208b have been paid great attention 18–31. Among them, miRNA-499, miRNA-208a and miRNA-208b belong to the same family named the miRNA-208 family. MiRNA-208a and miRNA-208b have identical nucleotide sequences of the seed region with only three different nucleotides in the rest of the sequences. MiRNA-208a is located in an intron of the Myh6 gene and is expressed in the heart, while miRNA-208b is in an intron of the Myh7 gene and is expressed in the heart and skeletal muscle 17–27. Our previous review has summarized the use of circulating miRNAs as ACS biomarkers in detail and also several other outstanding reviews have provided sufficient informative data 17–28. In Figure 2, we have given an overview of circulating miRNAs as the diagnostic biomarkers for ACS. Here, we will only focus on the novel findings published in the last 2 years.

The prevalence of ACS greatly increases by ageing, while the accurate recognition of AMI, especially NSTEMI in the elderly, is still challenging 29. In a very recent study, plasma miRNA-499-5p was reported to be comparable to cardiac Troponin T (cTnT) in discriminating NSTEM from controls and HF patients. The diagnostic accuracy of miRNA-499-5p was even higher than conventional and high-sensitivity cTnT (hs-cTnT) in differentiating NSTEM from acute HF patients with modest cTnT elevation at presentation 29. A meta-analysis of available studies regarding using circulating miRNAs for diagnosing AMI shows that despite the heterogeneity of settings and circulating miRNAs studies, the diagnostic performance of circulating miRNAs is globally comparable to highly sensitive troponin testing, indicating the promising feature of circulating miRNAs as diagnostic biomarkers for AMI 30.

Besides plasma and serum, a recent study explored the role of using urine miRNA-1 and miRNA-208 as the diagnostic biomarkers for ACS 32. In a rat AMI model, urine miRNA-1 was increased and peaked over 50-fold increase at 24 hrs, and then it returned to the base level at 7 days after AMI. In urine from AMI rat, miRNA-208 can be easily detected and it is undetectable in urine from healthy individuals. However, in AMI patients, a 60-fold increase in miRNA-1 urine level can be found, while very low levels of urine miRNA-208 can be found in only 25% patients, indicating that in urine, unlike miRNA-1, miRNA-208 might not be a suitable biomarker for AMI 32. This study is of great importance because large molecular protein biomarkers including CPK-MB, TnT and TnI are difficult to enter the urine, making miRNAs as a special biomarkers for AMI 32. However, it is unclear whether other cardiac/muscle-specific miRNAs including miRNA-133a, miRNA-133b and miRNA-499 can be an alternative similarly in serum or plasma.

### Non-specific cardiac and skeletal muscle miRNAs

For quite a long time, other miRNAs that were not cardiac or muscle-specific have been overlooked as most researchers only focus on using cardiac or muscle-specific miRNAs as biomarkers for ACS. Recently, much progress has been made especially with the whole-genome miRNA expression determination techniques.

MiRNA-328 is a ubiquitously distributed miRNA and has been reported to be involved in atrial fibrillation 33. MiRNA-328 in whole blood and plasma of AMI patients was significantly elevated by 16.1- and 10.9-fold compared with the controls 34. The AUC of miRNA-328 in whole blood and plasma was 0.872 and 0.81, respectively. Interestingly, no significant difference for miRNA-328 was found between patients with or without arrhythmia 34. Plasma miRNA-30a was highly expressed at 4, 8 and 12 hrs after the onset of AMI, while miRNA-195 was at 8 and 12 hrs 35. By contrast, let-7d was lower throughout the whole time-points in AMI patients 35. To discriminate AMI from healthy controls, the combined ROC analysis of plasma miRNA-30a, miRNA-195 and let-7d revealed an AUC value of 0.93 and 0.92 at 8 and 12 hrs after onset respectively 35. In addition, a decreased miRNA-126 was reported in AMI and a remarkable finding was that miRNA-126 and cTnI expression levels exhibited the same trend 36. Moreover, plasma miRNA-214 was also reported to be decreased in UA and AMI 37.

Besides the miRNA candidate approach, a whole-genome miRNA expression determination was checked in peripheral total blood samples of AMI patients 38. Among the identified 121 dysregulated miRNAs, miRNA-1291 and miRNA-663b showed the highest sensitivity and specificity. In addition, miRNA-30c and miRNA-145 levels correlated well with infarct sizes estimated by the release of cTnT. Moreover, a unique signature of 20 miRNAs that predicts AMI with a specificity at 96%, sensitivity at 90% and accuracy at 93% was identified by a novel self-learning pattern recognition algorithm 38.

## Prediction

At present, no golden soluble biomarkers can be used to accurately predict patients who are at risk of developing ACS 2,3. In a recent prospective single-centre study, the predictive value of circulating miRNAs including miRNA-1, miRNA-208a, miRNA-499, miRNA-21 and miRNA-146a as biomarkers for ACS was determined in 332 suspected ACS patients on their presentation to the emergency department 3. All miRNAs tested were significantly increased in ACS patients, even in those symptom onsets within 3 hrs or with initially negative hs-cTnT. Among these miRNAs, miRNA-1, miRNA-499 and miRNA-21 significantly improved the diagnostic value in all suspected ACS patients when added to hs-cTnT with an AUC of 0.9 3. Interestingly, these miRNAs were strong predictors of ACS independent of clinical variates including patient history and other cardiovascular risk factors. Moreover, the combination of these miRNAs led to a much higher AUC (0.94) than that of hs-cTnT (0.89) 3. This study indicates that miRNA-1, miRNA-499 and miRNA-21 can add predictive power to the established standard for ACS 3.

Besides the miRNA candidate approach, an alternative way combined miRNA arrays and qRT-PCRs was used in a prospective study on circulating miRNAs and risk of MI 39. The Bruneck study is a prospective, population-based study started in 1990 as an age- and sex-stratified random sample of all inhabitants of Bruneck 40–79 years old. The association of baseline levels of circulating miRNAs (year 1995) was explored with the incidence of MI over a 10-year period (year 1995–2005). In multivariable Cox regression analysis, miRNA-126, miRNA-223 and miRNA-197 were consistently and significantly related to incident MI with a hazard ratio of 2.69 (95% confidence interval: 1.45–5.01), 0.47 (95% confidence interval: 0.29–0.75) and 0.56 (95% confidence interval: 0.32–0.96) respectively 39. This is the first study showing that circulating biomarkers including miRNA-126, miRNA-223 and miRNA-197 could be used to identify patients who are at the risk of developing MI for a long period.

Cardiac death is a most serious complication after discharge for AMI 40. Considerable interest has been paid to whether a subset of circulating miRNAs was predictive of cardiac death after discharge for AMI 40. Through a high-throughput miRNA array and subsequently real-time reverse transcription-polymerase chain reactions (RT-PCRs), serum miRNA-155 and miRNA-380* were respectively four- and threefold higher in patients who had cardiac death within 1 year after discharge compared to those did not have cardiac death 40. Accordingly, these miRNAs might be predictive of cardiac death post-AMI, although the cause–effect relationship between the elevation of serum miRNA-155 and miRNA-380* levels and cardiac death post-AMI still needs to be clarified 40.

The number of ischaemic HF patients post-AMI is increasing. Novel biomarkers that can predict HF in post-AMI patients are highly desired for optimizing the therapy 41. In a small cohort study composed of 21 patients who developed HF within a year after AMI and 65 matched controls, serum miR-192 was found to be elevated in AMI patients who developed HF. Interestingly, as miR-192 is p53-responsive, the serum level of other two p53-responsive miRNAs, miR-194 and miR-34a, was also checked. miR-194 and miR-34a were found to be correlated well with LV end-diastolic dimension 1 year post-AMI, indicating that circulating p53-responsive miRNAs might be predictors for HF after AMI 41.

## Prognosis

The prognostic value of muscle or cardiac specific miRNAs including miRNA-1, miRNA-133a, miRNA-133b, miRNA-208a, miRNA-208b and miRNA-499 and hs-TnT was determined in a large ACS cohort 42. MiRNA-1, miRNA-133a, miRNA-133b and miRNA-208b were independently associated with hs-TnT as revealed by a multiple linear regression analysis. MiRNA-133a and miRNA-208b were significantly associated with the risk of death in univariate and age- and gender-adjusted analyses. Although after adjusting for hs-TnT both miRNAs lost their independent association with outcome, it is still possible that measuring these miRNAs at later time-points might enhance their prognostic value 42. Similarly, another study also found that increased miR-208b and miR-499-5p levels were associated with increased risk of mortality or HF within 30 days, but that the association was lost after adjusting for TnT 43.

In another study, the prognostic value of serum miRNA-133a in STEMI was determined, with the occurrence of major adverse cardiovascular events including death, re-infarction and new congestive HF within 6 months after infarction as a primary clinical end-point 44. It was found that major adverse cardiovascular events occurred more often in the miRNA-133a over a median group in ACS patients. Although miRNA-133a levels failed to predict clinical events independently in that study, significant correlations were shown with all prognostic-relevant cardiovascular magnetic resonance imaging markers including infarct size, microvascular obstruction and myocardial salvage index 44.

A very recent study inspired the confidence of developing circulating miRNAs as biomarkers for the prognosis of ACS 45. LV remodelling after AMI is associated with adverse prognosis 45. Circulating miRNAs associated with LV remodelling defined as increase in LV end-diastolic volume between discharge and follow-up after AMI were determined by microarrays and PCR in 90 STEMI patients. Using a system-based approach, miRNA-150 was found to be able to predict LV function and remodelling after AMI, which is even superior to Nt-proBNP, the golden standard biomarker currently used in the clinic 45. However, another report demonstrated that miR-133a and miR-423-5p failed to predict LV remodelling after AMI, although time-dependent increase of these two miRNAs was observed 46.

These preliminary studies suggest that circulating miRNAs hold a great potential to improve the prognosis of ACS patients who can benefit from swift initiation of treatment. Larger prospective studies are mandatory to elucidate the contribution of circulating miRNAs on top of established risk assessment strategies in ACS.

## Reaction to therapy

The identification of informative biomarker is an exceptionally valuable tool for helping physicians in choosing treatment options 1. Moreover, these biomarkers might pave the way from common treatment to individualized treatment, named personalized therapy 1,8. Several therapeutic options are available in ACS patients’ management 2. Unfortunately, some options have potential serious side effects and are costly as well 2. Therefore, identifying those who will benefit most from a particular therapy is extremely important. miRNA profiling is being considered for the prediction of response to a specific therapy 1–2.

Antiplatelet therapy is an essential therapy for ACS 1–2. Currently, assessing platelet responses *ex vivo* is a major test of platelet reactivity. Unfortunately, this approach fails to give any helpful guidance to antiplatelet medication 1–2. In a dose-escalation study of healthy volunteers, 92 miRNAs levels in plasma were checked at four different time-points 47. The groups divided in that study included the baseline without therapy, 1 week with 10 mg prasugrel, 2 weeks with 10 mg prasugrel + 75 mg aspirin and 3 weeks with 10 mg rasugrel + 300 mg aspirin. Results were confirmed with Taqman-based qPCRs in the same cohort and were also validated in an independent cohort of patients with symptomatic atherosclerosis receiving low-dose aspirin at baseline. Platelet miRNAs, including miRNA-223, miRNA-191, miRNA-126 and miRNA-150, decreased upon platelet inhibition in plasma 47. This study is the first one to identify plasma miRNAs responsive to antiplatelet therapy and gives a novel concept that circulating miRNAs can help provide a tailored effective antiplatelet therapy. Moreover, this study opens a window for the possibility of using circulating miRNAs as monitors of the efficiency of a specific therapy.

## Potential biological functions

**Figure 3:**
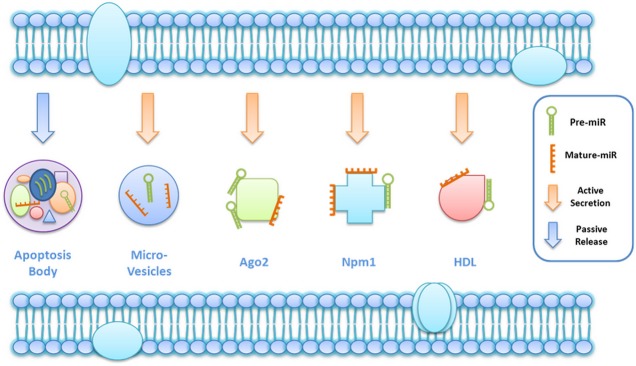
Heterogeneous forms of circulating microRNAs. Ago2: Argonaute 2; NPM1: Nucleophosmin 1; HDL: high density lipoprotein; miR: MicroRNA.

Despite the obvious potential of circulating miRNAs as ACS biomarkers regardless of diagnosis, prediction, prognosis and reaction to therapy (Table 1), it remains obscure whether circulating miRNAs have potential biological functions or not 20. It would be extremely interesting to determine whether the release of circulating miRNAs in ACS is an active secretion process or sorely a passive release as necrosis-associated biomarkers 18–48. In other words, it remains unclear whether circulating miRNAs are messengers in cell-to-cell communication or merely degradation products without any biological function (Fig. 3) 18–48. Interestingly, not all miRNAs highly expressed in the heart are released into the circulation during AMI 18–31, making the release of circulating miRNAs more likely to be an active process and these circulating miRNAs might have potential biological functions.

**Table 1:** Studies assessing circulating microRNAs as biomarkers of prediction, prognosis and reaction to therapy for acute coronary syndrome

Diseases	Controls	Dysregulated circulating microRNAs in diseases	Additional values than established biomarkers	References
Prediction				
ACS	Non-ACS	miR-1, miR-208a, miR-499, miR-21, miR-146 up-regulated	miR-1, miR-499, and miR-21 increased the diagnostic value when added to hs-troponin T	3
MI	Non-MI	miR-126 up-regulated; miR-223, miR-197 down-regulated	miR-223 and miR-197 showed negative associations, while miR-126 showed a positive association with subsequent MIs (traditional risk factors failed)	39
Cardiac death post-AMI	Event free	miR-155, miR-380^*^ up-regulated	Serum miR-155 and miR-380^*^ were respectively four and threefold higher in AMI patients who had cardiac death within 1 year after discharge	40
Heart failure post AMI	Event free	miR-192, miR-194 and miR-34a were up-regulated	miR-194 and miR-34a were found to be correlated well with LV end-diastolic dimension 1 year post-AMI	41
Prognosis				
ACS at risk of death	Event free	miR-133a, miR-499-5p and miR-208b up-regulated	After adjusting for hs-troponin T, both miRs lost their independent association with outcome	42
STEMI with major adverse cardiovascular events	Event free	miR-133a up-regulated	Significant correlations were shown with all prognostic relevant cardiovascular magnetic resonance imaging markers including infarct size, microvascular obstruction, and myocardial salvage index	43–44
LV remodelling after AMI	Event free	miR-150 up-regulated	Predict LV function and remodelling after AMI, which is even superior to Nt-proBNP	45
Reaction to therapy				
antiplatelet therapy in healthy volunteers and symptomatic atherosclerosis	Without antiplatelet therapy	miR-223, miR-191, miR-126, miR-150 down-regulated	Platelet miRs, including miR-223, miR-191, miR-126 and miR-150, decreased upon platelet inhibition in plasma	47

ACS: Acute coronary syndrome; AMI: Acute myocardial infarction; STEMI: ST-segment elevated myocardial infarction; miR: MicroRNA.

Circulating miRNAs are resistant to RNAase digestion and remain stable even in the RNAase-rich environment. In addition, they can also withstand repetitive freezing and thawing cycles, making them more attractive as biomarkers for ACS. This is because they are packaged in lipid vesicles or associated with protein complexes 17–28. Actually circulating miRNAs are heterogeneous as they exist both in non-vesicle form and in vesicle-associated form 10,48. Some circulating miRNAs are packaged into apoptotic bodies or microvesicles, while others are solely complexed with argonaute 2 (Ago2) protein, nucleophosmin 1 (NPM1) and high-density lipoproteins (HDL) 10,48. Microvesicles, also called exosomes or microparticles, contain more than 100 miRNAs and can be delivered from one cell to another 48–49. Thus, circulating miRNAs might function as intercellular or interorgan communication mediators 48–49. Circulating miRNAs might be delivered to recipient cells and thereafter regulate the translation of their target genes 48–49. A case in point is that microvesicles containing miRNA-150 were taken up by endothelial cells and regulated its migration at least partially through repressing its target gene c-Myb 50. However, it is difficult to totally rule out the contribution of non-miRNA-related pathways. In addition, although the idea of functional circulating miRNAs is intriguing, their relevance remains a matter of debate 48.

## Perspective

At the present stage, using circulating miRNAs as biomarkers for ACS is still in its infancy 18. Most works summarized in this review are single-centre studies with a limited number of patients. Thus, multicentre large-scale studies are highly needed to determine the potential of using circulating miRNAs as biomarkers for ACS.

qRT-PCR is the most widely used method for determining the level of circulating as it allows quantification of circulating miRNAs down to the level of copy number within a cell, potentially providing precise cut-off concentrations for diagnosis 24. However, many issues still exist in this field. First, proper endogenous controls for data normalization are still unclear 6–49. Some groups will use 5S or U6 as the endogenous controls, while others think that they are improper. Some groups will use miRNAs that appear most stable in the profiling procedure after global normalization, while others prefer to use spiked-in miRNAs, especially synthetic Caenorhabditis elegans miRNAs including cel-mir-39, cel-miR-54 and cel-mir-238 17,49. Interestingly, some groups also use the plasma volume as normalization (pmol/l) as they think that the amount of miRNAs per ml of plasma could be a standard method used in the clinic 32. Because of the huge difference in choosing endogenous controls, it is hard to compare the data presented by different groups. Thus, multiple endogenous controls are recommended to be employed to make sure of a robust finding regardless of the way of standardization 17,49. These can include spiking of synthetic miRNAs, global normalization and standardization to individual miRNAs that are similarly detected in all controls and are not found to be associated with diseases 17,49. Secondly, RNA isolation and subsequent quantification using real-time PCR as shown in most published work are time consuming 17. One major advantage that circulating miRNAs can offer compared with already established biomarkers is their relatively early release after myocardial injury. Thus, quickly available assays, ideally in the form of a bed-side test, need to be developed to allow broad utilization of circulating miRNAs as biomarker 4,17. Actually, the fast assay of RNA is a highly pursued research topic and much progress has been made including fluorescence and ELISA 4–10. In addition, electrochemistry might be an alternative choice 52. Any breakthroughs in this area will overcome the pitfalls in the near future. Thirdly, a better understanding of the source of circulating miRNAs will help fully identify circulating miRNAs as biomarkers for ACS. In a recent study, whether the heart is a source for circulating miRNAs in ACS has been checked by measuring the concentration gradients of miRNAs across the coronary circulation 53. A significant increase in the circulating levels of miRNA-499 and miRNA-133a across the coronary circulation was observed in troponin-positive ACS compared with patients of coronary artery diseases, suggesting that these two miRNAs are released into the coronary circulation during myocardial injury 53. However, miRNA-126 was found to be significantly decreased during transcoronary passage in ACS patients, suggesting that miRNA-126 is consumed during transcoronary passage 53. In addition, membrane-bound vesicles and vesicle-free but protein-protected protein-miRNA complex are also potential sources 30–54.

## Conclusions

Circulating miRNAs have emerged as novel promosing biomarkers for ACS including its role in diagnosis, prediction, prognosis and reaction to therapy. The concept that miRNAs can be actively secreted raises interesting biological questions, which might be relevant for cardiovascular disease. The value of circulating miRNAs as biomarkers should be re-evaluated when incorporating the traditional biomarkers into the current clinical model so that the way could be paved from the bench to the bedside. Considering the advantageous properties and the continuously increasing numbers of studies, circulating miRNAs definitely have the potential to be reasonable diagnostic tools once their infancy has passed.
